# ‘It’s the sense of responsibility that keeps you going’: stories and experiences of participation from rural community health workers in Guatemala

**DOI:** 10.1186/0778-7367-70-18

**Published:** 2012-08-10

**Authors:** Ana Lorena Ruano, Alison Hernández, Kjerstin Dahlblom, Anna Karin Hurtig, Miguel San Sebastián

**Affiliations:** 1 Division of Epidemiology and Global Health, Umeå University, SE-901 85, Umeå, Sweden

**Keywords:** Community health workers, Community participation, Guatemala, Primary Health Care, Alma-Ata declaration

## Abstract

**Background:**

In 1978, the Alma-Ata declaration on primary health care (PHC) recognized that the world’s health issues required more than just hospital-based and physician-centered policies. The declaration called for a paradigm change that would allow governments to provide essential care to their population in a universally acceptable manner. The figure of the community health worker (CHW) remains a central feature of participation within the PHC approach, and being a CHW is still considered to be an important way of participation within the health system.

**Methods:**

This study explores how the values and personal motivation of community health workers influences their experience with this primary health care strategy in in the municipality of Palencia, Guatemala. To do this, we used an ethnographic approach and collected data in January-March of 2009 and 2010 by using participant observation and in-depth interviews.

**Results:**

We found that the CHWs in the municipality had a close working relationship with the mobile health team and with the community, and that their positions allowed them to develop leadership and teamwork skills that may prove useful in other community participation processes. The CHWs are motivated in their work and volunteerism is a key value in Palencia, but there is a lack of infrastructure and growth opportunities.

**Conclusion:**

Attention should be paid to keeping the high levels of commitment and integration within the health team as well as keeping up supervision and economic funds for the program.

## Background

In 1978, the Alma-Ata declaration on primary health care (PHC) recognized that the world’s health issues required more than just hospital-based and physician-centered policies. The declaration called for a paradigm change that would allow governments to provide essential care to their population in a universally acceptable manner. In order to do this, communities and individuals needed to be more involved in health systems and health policies so that health services would be more responsive to local needs [1,2]. Participation at the individual level meant to involve community members as volunteer health workers and today community health worker (CHW) programs are a way to engage volunteer work from the communities in health promotion and disease prevention processes [1]. Being a community health worker remains a central feature of participation within the PHC approach, and being a CHW is still considered to be an important way of participation within the health system [3,4].

Community health workers can be defined as individuals with no formal or professional training, delivering basic health services in the context of an intervention [4]. As community members, they are selected by, and accountable to their community and are supported by the health system, even if they are not necessarily part of it [5,6]. CHWs have been described as ‘the cornerstone’ of underfunded health systems because they bridge together, community-level interests and health systems goals [3,7-9]. Studies show that successful CHW programs contribute to continuity of care and to increased compliance with treatments, even in isolated areas. They may improve communication levels between a health center and the population it provides care for by giving community members a voice and role in health promotion processes [3-7].

According to a recent Cochrane review [8], the use of community health workers has many proven benefits in a wide range of interventions that go from maternal and child health to tuberculosis control. However, these kinds of studies focus on the efficiency and efficacy of programs, and not on the lives and experiences that these community health workers have, and how that impacts community life. Other studies focus on the role that gender plays within these programs [9,10] or on how specific incentives can contribute to improving the sustainability of programs [11], but do this without trying to gain a deeper understanding of how the experiences of CHWs can provide information about how they relate with their communities and the health system. These human factors are crucial to the success of CHW programs in health promotion. In this paper, we explore how the values and personal motivation of community health workers influences their experience with this primary health care strategy in Guatemala.

Guatemala started implementing CHW programs in the 1960s as a way to respond to the health system’s inability to provide primary care to rural populations. Today there are two main types of CHW, with different levels of training and rosters of community-based services [12]. The first are known as *promotores de salud*, who began working in the 1960s and focused on preventive and curative medicine at the community level. Many were trained by the Ministry of Health (MoH) and other non-governmental organizations (NGOs), and their role as ‘little doctors’ was to diagnose and treat a wide range of diseases. Historically, the *promotores* actively engaged in preventive medicine, integrated development projects and social and political movements [12]. The MoH no longer trains *promotores,* and it is impossible to know how many are still working today. However, we do know that their community-level work mainly consists of small ‘clinics’ where they charge for the services they render.

The second type of CHW collaborates with the MoH through the Sistema Integral de Atención en Salud [integrated health care system] known as the SIAS. This program was implemented as a response to the Guatemalan state’s 1996 mandate to provide health care to previously excluded populations. The program provides decentralized care to rural populations through outsourced NGOs that deliver a package of services that cover maternal health, infant and child care, emergency medicine and environmental health to jurisdictions of about ten thousand people [12-15]. The SIAS works through mobile health teams made up of a physician or professional nurse, an auxiliary nurse and a rural health technician that make monthly visits to the communities. The team works with a team of volunteer CHWs in each of the communities in the jurisdiction, known as *facilitadores comunitarios*. The *facilitadores*’ main duties are to facilitate the team’s services to the community during their monthly visit, attend patients when the team is not present, identify cases for referral, maintain the census and epidemiological monitoring, and increase awareness of health issues. They participate in monthly capacity-building workshops and receive a first aid kit with over-the-counter medicines and receive a stipendium of about 50USD a month for fulfilling these responsibilities, to which they are expected to devote around four hours a day. They work directly under the nurse, who is in charge of their on-going training and supervises their work.

## Methods

### The setting

The municipality of Palencia, in the province of Guatemala, is located 18 km away from the capital city. Of the 55,410 inhabitants, 70% live in rural areas and about 38% is poor [16,17]. The main economic activity is small-scale coffee plantations and subsistence farming. Previous studies in this municipality show that Palencia is a tight-knit community that values solidarity and trust, and its community leaders feel supported by the municipal and health authorities [18]. This has allowed them to develop close working relationships between these authorities and the community leaders report feeling a deep commitment when it comes to community participation over long periods of time [19].

The MoH in Palencia has a relatively well-developed infrastructure that allows 32 of the 49 communities to access either the health center or one of the seven health posts easily. For the 17 communities located more than two hours walking distance away, the SIAS has contracted one NGO to provide care through a network of twenty CHWs that act as *facilitadores de salud* for the health team.

### Data collection

Data collection for this paper was carried out between January-March of 2009, and January-March of 2010. During this time, the first author joined the health team in their visits to all the communities in the NGO’s jurisdiction, and went to each community on at least two occasions, she also participated in two of the monthly training sessions conducted in Palencia’s health center. Field notes and transcripts of interviews made up the core data, which was gathered through participant observation, in-depth interviews and informal, personal communications between the first author and the members of the health team. This was done as part of a larger study on the role of social participation in municipal-level health systems that is part of the first author’s doctoral thesis.

Using purposive sampling, several informal interviews and eighteen in-depth interviews were conducted with two of the members of the health team and with sixteen *facilitadores comunitarios*. In Palencia, most of the *facilitadores* were female, so the thirteen interviews with female *facilitadores* and three with male ones reflected the actual gender distribution of the CHWs in this municipality. Of all sixteen *facilitadores*, only four reported finishing high school. Of those four, one had become a teacher and the other underwent training to become an auxiliary nurse, but had never worked as one. As with most people in Palencia, their main economic activity was agriculture and some had a small community pharmacy to supplement their income as well. In total, twelve of the sixteen *facilitadores* had been with the program for more than four years.

For the in-depth interviews, an interview guide focusing on the experience of being a CHW was used. The guide dealt with how the *facilitadores* got involved with the SIAS, what kind of training they had received and the work they carried out. We also discussed family support and personal interests, and their plans for the future. Seventeen out of the eighteen interviews were tape recorded with permission from the informant. Most of the data for this study was obtained by using in-depth interviews and discussions. However, the first author was a participant observer and gathered rich information through the writing of field notes. By using un-structured observation, a more complete social setting was captured. Observation is a dynamic activity that is more likely to produce evidence regarding processes than only using in-depth interviews [20,21]. These observations were written down daily, and later provided contextual information for the carrying-out of the thematic analysis.

### Data analysis

The aim in applied ethnographic research is to study a particular situation or context in order to find the constructs, structures and phenomena that constitute a dynamic social process in a way that allows comparison with social groups that might be similar or very different [22,23]. Comparability and translatability are analog to the positivist construct of generalizability and validity, and they allow an ethnographer to gather data that provides a depth of understanding that would be impossible to gain through any other method [22].

By using thematic analysis, we identified themes characterizing the experiences of being a CHW in Palencia [24]. Thematic analysis allows researchers to group codes and categories that are similar into themes that reflect specific patterns in the data [22]. This happened through the process of careful reading and coding of the data into meaning units grounded in the text [25]. In our analysis, we identified emerging and a priori codes that were part of our interview guide. We summarized transcripts and outlined key points in the interviews, coded using the open code software program and identified categories through an interactive process between the researchers. The categories were then linked into themes and later corroborated by close scrutiny of the analysis [24,26]. The extensive field notes also provided rich information for the context of the interviews and served as additional documentation of the informal conversations between the first author and the *facilitadores*/health team.

### Ethical considerations

In Guatemala, it is only necessary to ask for ethical clearance when conducting clinical trials or human testing. However, we procured ethical clearance with the local municipal authorities, with the MoH and with the community health workers in our study. We did this by presenting our project and our methodology to all the participants. We obtained verbal informed consent from the interviewees and informed them that they could withdraw at any time without any consequences. We asked permission to tape record the interviews or to take notes, and guaranteed anonymity to all participants. In the findings, we used pseudonyms to protect our informant’s identities. We later shared the results with all the informants.

## Results

We have characterized the experience of being a community health worker in Palencia into three themes: getting started, the motivation required for the job and finally, the work of a CHW.

### Theme 1: Getting started

There were four different ways of getting involved as a *facilitador comunitario* in Palencia. The most common case in this municipality was to be asked by the community to ‘step-up’ to the position. This was the case for many of the municipality’s *facilitadores*, who were asked to participate when the SIAS program started to work in the area. Mario was a leader in his community and was involved in other committees or initiatives before starting his work with the *SIAS* for his community. He had a long story of participation and leadership before deciding to focus solely on health-related issues. He told us:

"*I started because the community elected me and I accepted because I am very committed to my community and have been since the very beginning… I am the president of the development committee, I am a leader in my community and like I told you, I am here working and I will work until there is no more work to be done.*"

Sometimes, it was the health team and not the community who approached a community member with the request to work with them. This was the case for Ana, who started to work after receiving encouragement from the doctor to join the team and act as their community counterpart. This doctor supported her when going back to school and helped Ana get the necessary skills to become a *facilitador*:

"[The doctor] *said she could tell I was very smart and asked me if I wanted to work with her. I told her that I couldn’t read or write, so I probably couldn’t do the job. She told me that the job was a means to learn and so I started going to night school. She gave me the job and helped me to learn what to do and today I am still here.*"

Support from family members was an important factor when deciding to become a *facilitador* and parental and spousal support often played a major role in making this decision. Female *facilitadores* reported having the support of their spouse and the encouragement of their family members, and two of the three male *facilitadores* joined the program after many months of helping their wives with their workload. Their help included walking the few hours to Palencia’s health center to retrieve drugs or supplies, or assisting their wives in daylong, tiresome activities like child-growth monitoring. As Doña Carolina from Pie del Cerro explains, her husband Mario got officially involved after years of supporting and helping her do the work:

"*I started when the health center invited me to participate, and I wanted to do it but I was pregnant at the time. So I worked as long as I could and then my sister and my husband helped me when I was too big. My sister stayed on even after I had the baby but then couldn’t help me anymore and my husband continued to help me with small things. Then the doctor asked me if I wanted him to officially help me, because he contributed so much already. That’s how he got the job.*"

Finally, the fourth way to become a *facilitador* is to inherit the position. This was the case for Susana, who took the job after her mother had passed away. For her, continuing her mother’s work was a way to pay tribute to her memory and it contributed to what she was already doing as the village’s teacher. By doing both jobs, she felt as if she could help the children and their mothers more. She told us:

"*Working on both things (the school and the SIAS) gets me more support. It’s very important because the kids’ mothers look for me and know where to find me. Working with the school and this has shown me that people working together can improve things, and that deserves a lot of respect.*"

### Theme 2: The motivation behind the work

For some of the *facilitadores*, their values and personal characteristics of leadership and commitment to learning were channeled into health by the desire to become a health professional. They expressed that from a young age, they had wanted to become nurses or physicians. However, negative experiences with the educational system when they were young, their families’ lack of funds to pay for nursing or medical school and the required travel made it impossible for them attain this particular goal. Through positive encounters with a doctor or a nurse, they were introduced to, and motivated to do primary care work. Out of these relationships, the *facilitadores* received one-to-one training and encouragement to learn. This made them feel special and allowed them to start working with health at the community level. Carolina, a woman who always had a desire to become a nurse herself, recalled how a nurse in the health post near her house helped get her into a study plan to become a *promotor de salud* and provided extra training:

"*I started to work in this when I was about 15, there was a nurse in the community where I grew up that was very nice to me. I would go to the health post to help clean and that’s how I met her. Eventually there was a group of girls that wanted to learn how to inject patients, I was in that group, and that’s how I started to work. I learned how to inject and I would help to clean up the post voluntarily… I was there once a week and when sick or hurt people would come, this nurse would tell me to go with her and I would. That’s where I got practice. I learned to suture, put in an IV… That was a very nice experience… no one else there learned how to do all that but I did because I stayed with the nurse, voluntarily. She would say ‘let’s do it together’, I liked it, and I paid attention and helped her with the scissors or with other things. [After showing me] she would let me do it and that’s how I learned… Everyone knew about me so when the SIAS started to work in 1996 or 1998 the community elected me and here I am, working still. I have learned a lot more with the training the SIAS gives us too.*"

The *facilitadores comunitarios* in Palencia feel that working together and giving their time to the community is the only way to achieve a better quality of life for everyone. By sharing their time and expertise, they can help others. These values are at the core of family and community life and give the *facilitadores* a sense of purpose and motivation to continue, even when the work piles on top of their other family and work responsibilities. Irina, who was chosen to be a *facilitadora* by her community, and who has a long experience of leadership in her parish church, explained:

"[People] *call me because they want me to help and I always do… it is my responsibility to do a good job because if you want what is best for your family, for your children, you also want what is best your community. That is my goal in life, to see my children grow and learn more and I tell them that participating is the way to do it. That is my purpose in life and I have served since 2004. It’s just nice to help and teach others so that everyone knows what to do and how to help.*"

As *facilitadores* work with their communities, they develop a sense of commitment and responsibility to deal with the negative aspects of the work. Some of the *facilitadores* reported that the workload can get too heavy at times, and that time spent outside the home trying to deal with community members that expect payment, drugs or some manner of healthcare in return for allowing their children to be weighed or vaccinated can be tiring. Sometimes, the lack of support from official community committees or from stakeholders in the municipality can make the work seem overwhelming. Aurora has worked as a *facilitadora* for eight years, with her husband helping her for the last four. She told us how they deal with the lack of support and the weariness that can come from the job:

"*You feel the responsibility to serve because it starts like something you volunteer for and then you see it as something ‘you have to do’. It’s like a duty and you just have to do it. That motivates you, to see it like a responsibility you have so that when you come home from your normal work and are tired you keep going. It’s the sense of responsibility that keeps you going.*"

### Theme 3: The work of a CHW

There are specific tasks that *facilitadores comunitarios* have to do as part of their jobs in the SIAS and in Palencia, all the *facilitadores* reported having clearly outlined responsibilities. Knowing exactly what was expected of them from the SIAS and from the community meant they could take on the responsibility without the fear of over committing their time or of not being able to do the job. This has allowed them to develop close relationships with their community, which has a direct impact on how they carry out and feel about their work. Isabel is a 20-year-old that inherited the position from her sister. Counting her, there have been four *facilitadores* in her family. For her, being a *facilitadora* was a way to get closer to her community. She reflected on her feelings and said:

"*I am very close to the families I work with. I like how they treat me and how responsible they are when it comes to taking care of their children. All I have to do is to tell them we need to do something and they do it, even if they are busy with their housework. They know this is important and they let me work. They tell me that they will miss me when I leave the position… and I think this experience is really good and that it will help me later… I like working here.*"

Having an active relationship based on partnership and mutual respect and trust is at the core of the work dynamic between the health team and the *facilitadores*. Palencia’s *facilitadores* and the health team described their interactions as open, flexible and mutually satisfactory. Their relationship was based on respect and on knowing the importance of each one's role within the team. The feeling that their positive outcomes stem from their teamwork and viewing their working relationship as a collaboration has provided an opportunity for both sides to learn and grow. This has contributed to creating a good work environment that promotes personal growth. The health team doctor told us:

"*Their work is very important; it helps me as a doctor so that I have information [about the people in that community] there when I need it. [The facilitadores] are definitely important because if they are not here, if they are not leaders, then all of our work come up short. That is their main role, to be leaders and to help, so we all work together.*"

Esmeralda started working as a *facilitadora* after being asked by Ana. Through her work she has been able to learn both through experience and by working together with the health team:

"*I like this job because we learn new things, we learn them in a clear way so that stuff that used to be kind of blurry, things we only used to hear others talk about, we understand now. We do that because the doctors and nurses tell us about it, they talk with us and that is really good. It makes me happy to know these things and to work with them, and I will keep at it until it’s time to take my leave.*"

Finally, the work that the *facilitadores comunitarios* do was often seen as a chance to develop their leadership skills and to improve community life. This is because for them, becoming a *facilitador* was synonymous with becoming agents of change. Mario reflected:

"*I like to work with children and see them grow and be happy. I give them vitamins and I check on them and talk to their mothers so everyone understands… now more people come to see the doctor, accept the vaccines. You have to go out and see what needs the community has and do something about it. That is why you are a facilitador.*"

In general, the *facilitadores* felt that they were doing more than just ‘going to work’. For most of them, the job was part of a larger process of community participation and leadership. Some had been involved in other projects before and some started to get involved because of this position. Many of them see it as an experience that will let them continue their community work, much like Mario:

"[Being a facilitador comunitario] *helps a lot because it gives you experience and each experience helps you in the future. If you start working in this and you already have experience from another project, another job, then that helps you. If I know how to be a leader and how to be a facilitador, then I can help with so many things.*"

The skills *facilitadores* acquire also help them to plan for their future. Some of them wish to keep on serving their communities, and see their job as a part of a larger scheme that leads to a better life for all the community. The work the *facilitadores* do lets them learn more about the responsibilities that community leaders need to have. To accept the position of *facilitador comunitario* requires the development of leadership skills because once other community members and leaders recognize the work the *facilitadores* do, they will try to get them involved in other areas. Like Carolina said:

"*People look me up because once they saw me as a leader; they always try to get me involved in everything. I give the community information and I help in any way that I can. No problem for us to do it, to help a little.*"

## Discussion

In Palencia, the *facilitadores* seem to be able to bring new voices and perspectives to community issues through the opportunities that their work with the SIAS brings. Their experience as CHWs has provided a means of contributing to the development of the community. Our findings show that the CHWs in this study seem to be satisfied with the work they are doing and the role assigned to them by the SIAS because it has allowed them to do work that they feel is meaningful and that fulfills their responsibility to the community. Because these CHWs integrate their work into their identity and see what they do in light of larger community processes, they have been able to create strong links between the community and the health system. In this section, we discuss the process of becoming, working and participating as community health workers in Palencia.

Previous studies have shown that one of the main components of successful community health worker programs is the ability to work closely with the communities they are serving [6-8]. One of the ways by which CHWs schemes do this is by including local, community-chosen staff [5]. However, the experience of Palencia’s CHWs show that initiation through community nomination is not the only way to find appropriate candidates. Familial relations provide potential applicants that know the job requirements and that have a shared vision of providing for the community. In addition, having input from the health team allows for the selection of individuals that fit in well with the team’s chemistry. These alternative routes to becoming a CHW help to bring in candidates that have strong ties to the work of community development through health promotion. In Palencia’s case, the individuals’ path to becoming a CHW shows how they are all part of a tight social network at the community level, and how that network takes interest in its own development [19]. This leads to greater leverage in the community’s dealings with health and municipal authorities.

In regards to the way the CHWs and the health team work, Lieu et al [27] found that having clearly outlined responsibilities and communication channels contribute to improving the quality of the work carried out at the community level. The rapport and consequent bond between the health team and the CHWs is organic, and rises from the uniqueness of the context, interaction of personalities and the way these individuals work together to respond to community needs in a flexible manner [28]. The relationship between Palencia’s CHWs and the health team was described as open, flexible and based on mutual respect. This leads to a strengthening of the services provided, to more referrals and to a clearer integration of the work between the project and the community staff. Finally, having clear mechanisms help CHWs develop realistic expectations of the commitment that is required of them, which is a key factor in retaining community staff [29].

Having committed community health workers that feel connected to the people they serve is a cornerstone of the CHW model [30]. By working directly with families, CHWs are able to develop unique relationships with both the community and the health system, something that makes them the center of coordination for community-level work [7,29,31]. In Palencia, the feelings of connectedness reported by the informants stem from shared community values that place special importance on community life and volunteerism [19], and from their personal characteristics such as dedication, reliability, persistence and the ability to earn and maintain trust [29]. Through this value set, CHWs develop a service attitude and obtain motivation to continue to work even in negative or complicated situations.

As a result of their work as CHWs, individuals gain knowledge, experience and skills that allow them to feel personal satisfaction and empowerment from their positions and daily activities [29,32]. By learning how to manage responsibilities and to work closely with the health team and their neighbors, the CHWs in Palencia were able to develop leadership and teamwork abilities. CHWs are often regarded as leaders in their communities because of their skills and knowledge, as well as for their ability to be flexible and respond to the different challenges of community work [28].

Because of their capacity to provide care for populations that are geographically or culturally isolated, health systems have recognized the importance of community health worker programs for many years. Superimposing CHW programs on weak health systems or placing undue burden on them often leads to failure. Community health worker programs usually suffer from inconsistent sources of funding resulting in high attrition rates from staff and community volunteers [29]. On a personal level, many CHWs find that there are few opportunities for advancement because of a lack of educational programs and institutional recognition of the position and work the CHWs do. In many programs, this has led to many CHWs either quitting or staying in their position because they feel obligated. The case of Palencia demonstrates how a CHW program resulted in predominantly positive and satisfying experiences for these community members, who felt their work allowed them to make a valuable contribution to their communities. Palencia does not represent a ‘typical’ Guatemalan municipality as it is located in a primarily urban area and has no ethnic minorities. We believe however that the study offers insights on important aspects of the interactions between CHWs, the health system and the community. The study only included voices from the CHWs and some health workers, and did not include experiences of community members. The perspective of the community could have highlighted another picture regarding the role of the CHWs. Further research is needed regarding the quality of service and care that these CHWs provide and regarding the overall performance of the program.

## Conclusion

Currently, the community health workers in Palencia provide the kind of voluntary work that is needed to deliver care and health promotion initiatives to rural and isolated populations in the municipality. They do this by building close working relationships based on respect and trust with the mobile health team and with the communities they serve. By working as volunteer, community-based staff, the CHWs contribute to strengthening the ties between the health district and the population. They are able to use their positions to develop leadership and teamwork skills that translate to other areas of community work and participation process.

Community volunteerism is an important component of the PHC approach, and in Guatemala the CHW program has proven useful in improving coverage levels in rural areas. Attention should be paid to the health system conditions that foster high levels of commitment and integration with the health care team. Providing more structured training and professionalization opportunities may contribute to CHW motivation as well as improving the quality of care available in rural areas. Supportive relationships with the health care team should provide opportunities for expanding skills and knowledge, and the CHWs’ contribution to the team’s work should be recognized and valued.

The feelings of satisfaction that these community health workers obtain from their work stems from personal and community values around the importance of participation, volunteerism and group work. Their communities are characterized by active local involvement in many areas of community development, which promotes a sense of responsibility to contribute through individual and collective efforts. This provides the intrinsic motivation needed to overcome the constraints of their positions, namely a lack of medical and administrative resources and infrastructure. Explicit attention is needed to support the development of community structures, specifically the development of councils and committees that can provide opportunities for meaningful participation in the different spheres of community life, not just in health.

## Competing interests

The authors declare we have no competing interests.

## Authors’ contributions

ALR conducted the fieldwork and drafted the first version of this manuscript. ALR, KD, AH, AKH and MSS analyzed and interpreted the data. All the authors read and approved the final version of this paper.
